# Periodontal biomarkers in cardiovascular disease: mechanisms, diagnostics, and clinical implications

**DOI:** 10.1007/s15010-026-02778-y

**Published:** 2026-03-19

**Authors:** Max Foroughi, Keykavous Parang

**Affiliations:** 1 Center for Targeted Drug Delivery, Department of Biomedical and Pharmaceutical Sciences, Chapman University School of Pharmacy, Harry and Diane Rinker Health Science Campus, 9401 Jeronimo Rd, Irvine, CA 92618 USA; 2 Department of Preventive and Restorative Dentistry, Arthur A. Dugoni School of Dentistry, University of the Pacific, San Francisco, CA USA

**Keywords:** Biomarkers, Cardiovascular diseases, C-reactive protein, Interleukin-6, Myeloperoxidase, Periodontitis

## Abstract

**Purpose:**

Periodontal disease is a chronic inflammatory condition increasingly associated with cardiovascular disease (CVD) beyond shared risk factors. This review evaluates evidence linking periodontal inflammation to cardiovascular outcomes and identifies periodontal biomarkers with potential relevance for cardiovascular risk assessment.

**Methods:**

A narrative review was conducted using PubMed, Scopus, and Web of Science databases, including studies published between 1990 and 2025. Epidemiological, clinical, and mechanistic studies examining associations between periodontal disease, inflammatory and microbial biomarkers, and cardiovascular outcomes were included. Biomarkers were categorized as inflammatory cytokines, acute-phase proteins, oxidative stress markers, lipid mediators, and microbial indicators. Evidence regarding periodontal therapy and emerging point-of-care diagnostic technologies was also reviewed.

**Results:**

Epidemiological studies demonstrate increased risks of coronary heart disease, stroke, and atherosclerosis in individuals with periodontitis, independent of traditional cardiovascular risk factors. Mechanistic evidence indicates that periodontal pathogens and host immune responses promote systemic inflammation, endothelial dysfunction, and atherogenesis. Key biomarkers associated with cardiovascular outcomes include cytokines (IL-1β, IL-6, TNF-α, IL-17), acute-phase proteins (C-reactive protein, fibrinogen), matrix-degrading enzymes such as active matrix metalloproteinase-8, oxidative stress markers (myeloperoxidase, malondialdehyde), lipid mediators (lipoprotein-associated phospholipase A2), and microbial markers such as antibodies to *Porphyromonas gingivalis*. Periodontal therapy has been associated with reductions in systemic inflammatory markers and improvements in endothelial function, although large, randomized trials remain limited.

**Conclusion:**

Periodontal biomarkers reflect biologically plausible mechanisms linking oral and cardiovascular inflammation and may enhance cardiovascular risk stratification. Further standardization, validation, and interdisciplinary collaboration are required for clinical translation.

## Introduction

The supporting structures of the teeth are affected by a chronic inflammatory disease, periodontal disease, which involves the periodontium (gingiva, periodontal ligament, and alveolar bone). The process begins with the formation of microbial biofilm, which leads to an uncontrolled host inflammatory response that damages the tissues and results in tooth loss [1]. The local effects of periodontitis are accompanied by a well-established connection to cardiovascular disease and diabetes, adverse pregnancy outcomes and Alzheimer’s disease and cancers and rheumatoid arthritis, and respiratory infections [2–7]. The low-grade systemic inflammation associated with periodontitis has been increasingly recognized as an important factor responsible for the oral–systemic connections elsewhere [8].

Epidemiological studies based on over three decades of research show that periodontitis patients have a higher risk of developing cardiovascular diseases, including coronary heart disease and stroke, and atherosclerosis [9]. The studies show how these inflammatory periodontal infections develop a systemic inflammatory disease through a new narrative review and identify diagnostic markers and tools to monitor these oral-to-systemic connections [10]. Large prospective cohort studies and meta-analyses have shown that this relationship is independent and persists free of the more classic risk factors, smoking, diabetes, and hypertension [11, 12]. The study by Blaizot et al. [13] by meta-analysis, found that patients with periodontitis were shown to have a 20% higher risk of having cardiovascular events in their future [13]. Studies have shown that periodontal pathogens and their products can be found in atherosclerotic plaques. Showing support for this biological pathway that exists between periodontal pathology and cardiovascular pathology [14]. The research indicates that periodontitis exists as a disease of the oral cavity, but at the same time affects vascular health throughout the body [15].

In addition to epidemiological data, biological mechanisms provide a strong rationale for this association. Bacterial pathogens of periodontitis and their virulence factors can cause systemic inflammation, bacteremia, and immune cross-reactivity, all of which are important contributors to vascular injury and atherogenesis [8, 14, 16]. Experimental models of atherosclerosis demonstrate that inoculation with *Porphymonas gingivalis* increases atheromatous development, while clinical trials show that successful periodontal therapy reduces systemic markers such as C-reactive protein (CRP) and interleukin-6 (IL-6), thereby supporting a biologically plausible association [17, 18]. In addition, the global burden of periodontitis is great. Nearly half of the adults of world suffer from periodontitis, and severe forms of it affect 10% of the population [19]. In view of the common prevalence of cardiovascular disease that exists (the major cause of mortality in the world), the understanding of this relationship and the targeting of it has considerable public health implications [20]. To ensure an understanding of and clinical applicability of this connection, reliable biomarkers are essential. For example, the measurement in blood or oral fluids of inflammatory mediators (e.g., IL-6, tumor necrosis factor-α (TNF-α)), acute-phase proteins (e.g., CRP), matrix-degrading enzymes (e.g., active matrix metalloproteinase-8 [aMMP-8]), and oxidative stress markers (e.g., myeloperoxidase), all of which should lead to an understanding of the systemic burden of inflammatory mediators and vascular dysfunction [18, 21]. Furthermore, the presence in the vascular tissues of microbial metabolism (e.g., lipopolysaccharides or their derivatives, and their DNA) gives support to the argument for an actual microbial contribution to atherogenesis [13]. The advent of omics approaches such as proteomics, metabolomics, and microbiome studies has increased consideration of the number of allowable candidate biomarkers and, as a result, increased interest in their possible contributions to precision diagnostics [22, 23]. The use of periodontal markers within models determined risk for cardiovascular disease shows promise in regard to improved early diagnosis, more accurate prognostic evaluation, and the possibility of point-of-care (POC) diagnostics being achieved [24, 25].

The purpose of the review is to critically review the current information as it pertains to the role of markers from periodontal tissues as they relate to cardiovascular disease, with special consideration to the mechanisms, diagnostic utility, and clinical implications. Thus, it is intended that, by integrating the current data, the possibility of marker-derived methods be achieved. Evidence for the early capture of disease, the refinement of the risks of disease, as well as personalized approaches to prevention and treatment of cardiovascular disease can be outlined.

## Methods (review design)

This review was presented as a narrative synthesis of current evidence investigating the association between periodontal biomarkers and cardiovascular disease. To reduce bias and improve transparency, a structured literature search was performed on the following electronic databases: PubMed/MEDLINE, Scopus, and Web of Science. Search terms included a combination of: “periodontal disease”, “periodontitis”, “cardiovascular disease”, “atherosclerosis”, “myocardial infarction”, “stroke”, “biomarkers”, “cytokines”, “C-reactive protein”, “myeloperoxidase”, “oxidative stress”, “microbial DNA”, and “point-of-care diagnostics”.

A structured literature search identified 312 records from the electronic databases (PubMed, Scopus, and Web of Science) initially. On removal of 78 duplicates, this resulted in 234 titles and abstracts screened for relevance. Of these, 128 full-text articles were assessed for eligibility based on predefined inclusion criteria. Following exclusion of studies that did not have cardiovascular endpoints, biomarker data, or sufficient methodological detail, 74 articles were identified as fulfilling these inclusion criteria, and it was these articles that were used in the final synthesis.

Inclusion criteria were:

1. Clinical, epidemiological, or experimental studies evaluating periodontal biomarkers in relation to cardiovascular outcomes or mechanisms.
2. Reviews, consensus statements, or meta-analyses that synthesized evidence on this relationship.
3. Studies reporting biomarker measurement in biological fluids (serum, plasma, saliva, gingival crevicular fluid) or cardiovascular tissues.

Exclusion criteria were:

1. Non-peer-reviewed reports, case studies with < 10 participants, or conference abstracts without full publication.
2. Studies without clear biomarker outcomes related to cardiovascular endpoints.
3. Animal studies that were not directly translatable to human mechanisms.

Evidence extracted was arranged thematically under inflammatory cytokines, acute-phase proteins, markers of oxidative stress, lipids and adhesion molecules, microbial/immune biomarkers, mechanistic pathways, and diagnostic applications. Findings were subjected to qualitative synthesis due to the heterogeneity of biomarker assays, study populations, and study designs.

## Types of periodontal biomarkers relevant to cardiovascular diseases

The periodontal biomarkers reflect the wide variety of molecules that result in both mechanistic and diagnostically relevant information about the oral–systemic cardiovascular relationship. Periodontal biomarkers include inflammatory cytokines such as IL-1β, IL-6, and TNF-α, which mediate local tissue destruction and perpetuate systemic inflammation; acute phase proteins such as CRP and fibrinogen, which are strong indicators of cardiovascular risk; oxidative stress markers such as MPO, malondialdehyde (MDA), and 8-hydroxy-2'-deoxyguanosine (8-OHdG), which mediate vascular damage and plaque instability; endothelial adhesion molecules (such as intercellular adhesion molecule (ICAM-1) and vascular cell adhesion molecule 1 (VCAM-1) that are involved in leukocyte recruitment and atherogenesis; as well as microbial or immune-related markers (Porphyromonas gingivalis antibodies, bacterial lipopolysaccharides, and DNA found in arterial plaques). Taken together, the periodontal biomarkers reflect the multiple pathways in which periodontal disease can affect cardiovascular disease. A summary of the major biomarkers, their biological significance, methods of detection for the biomarkers, and relevant references supporting their use are shown in Table 1, showing their potential value in research, diagnosis, and clinical risk stratification of patients.

**Table 1:** Local oral biomarkers and systemic biomarkers relevant to the periodontitis–cardiovascular disease axis

Biomarker	Class/type	Biological significance in periodontitis → CVD	Sample matrices	Detection methods (lab & POC)	Key Refs.
A. Local oral biomarkers *(primarily useful for diagnosis of periodontitis, disease activity, and monitoring local tissue destruction)*					
IL-1β	Inflammatory cytokine	Drives local tissue destruction; promotes systemic inflammation and endothelial activation	GCF, saliva, serum	ELISA, multiplex bead immunoassay; emerging electrochemical immunosensors	[1, 18]
IL-17	Inflammatory cytokine (Th17)	Neutrophil recruitment augments vascular inflammation and plaque progression	GCF, saliva, serum	ELISA/multiplex; flow cytometry (cellular sources)	[26]
TNF-α	Inflammatory cytokine	Amplifies inflammatory signaling; contributes to endothelial dysfunction	GCF, saliva, serum	ELISA/multiplex; immunonephelometry (research)	[1, 18]
MMP-8 / aMMP-8	Collagenase (neutrophil-derived)	Active connective tissue degradation in periodontitis; associated with systemic inflammation and cardiovascular risk	GCF, saliva, serum	ELISA, activity assays, POC chairside immunoassay	[27–29]
Matrix metalloproteinase-9 (MMP-9)	ECM-degrading enzyme	Cap thinning and plaque destabilization; elevated in periodontal inflammation	Serum/plasma; saliva/GCF	ELISA; zymography; activity assays	[30]
8-OHdG	Oxidative DNA damage marker	Reflects oxidative stress burden; associated with vascular injury and plaque vulnerability	GCF, saliva, serum	ELISA; HPLC-ECD/LC–MS	[31]
Composite salivary panels (e.g., IL-1β/IL-6/MMP-8)	Multi-analyte signatures	Higher diagnostic/prognostic yield than single markers; feasible in chairside screening	Saliva/GCF	Multiplex immunoassay; lab-on-chip; LFA prototypes	[2, 32]
Bacterial LPS (endotoxin)	Microbial component	TLR2/4 activation → systemic inflammation and endothelial dysfunction	Serum/plasma	Limulus assay; ELISA; endotoxin activity assay	[33]
B. Systemic biomarkers *(more relevant to systemic inflammation, vascular injury, and cardiovascular risk assessment/prognosis)*					
IL-6	Inflammatory cytokine	Upstream cytokine inducing hepatic CRP; associated with atherogenesis and adverse events	GCF, saliva, serum	ELISA/multiplex; high-sensitivity platforms; POC immunoassay prototypes	[1, 26]
CRP; hs-CRP	Acute-phase protein	Integrator of systemic inflammation; strong predictor of MI and stroke; elevated in periodontitis	Serum; saliva (research)	High-sensitivity immunoturbidimetry; ELISA; lateral-flow POC strips	[18, 34, 35]
Fibrinogen	Acute-phase protein/coagulation	Increase blood viscosity and thrombosis risk; elevated in periodontitis and CVD	Plasma	Clauss assay; immunoassay	[18, 36]
Serum amyloid A (SAA)	Acute-phase protein	Tracks acute-phase response; linked to vascular inflammation and lipid remodeling	Serum	ELISA; nephelometry	[37]
Myeloperoxidase (MPO)	Oxidative enzyme (neutrophil)	Generates reactive oxidants → LDL oxidation, endothelial injury; prognostic in ACS; elevated in periodontitis	Serum/plasma; saliva/GCF (research)	ELISA; enzymatic activity assays; electrochemical biosensors (research)	[38, 39]
Malondialdehyde (MDA)	Lipid Peroxidation product	Index of oxidative damage; correlates with periodontal inflammation and atherogenesis	Serum, saliva	TBARS/ELISA; LC–MS (advanced)	[40, 41]
Lipoprotein-associated phospholipase A2 (Lp-PLA2)	Lipid enzyme (LDL-bound)	Hydrolyzes oxidized phospholipids → LPC/oxFA; promotes plaque inflammation; elevated with periodontitis	Serum/plasma	Activity/mass immunoassays	[18, 42]
ICAM-1 (soluble)	Endothelial adhesion molecule	Leukocyte adhesion/transmigration; marker of endothelial activation; predicts events in CAD	Serum/plasma	ELISA; multiplex	[43]
VCAM-1 (soluble)	Endothelial adhesion molecule	Monocyte recruitment to intima; associated with inflamed/unstable plaques	Serum/plasma	ELISA; multiplex	[43]
*Porphyromonas gingivalis* antibodies	Microbial/immune biomarker	Serologic exposure marker; predicts stroke/CVD in cohorts	Serum	ELISA	[44]
Bacterial DNA (oral pathogens)	Microbial nucleic acids	Direct microbial signature in atherosclerotic plaques; supports invasion/persistence	Arterial plaque, blood	qPCR/16S sequencing; FISH	[14, 44]
Heat-shock protein 60 (bacterial/host)	Molecular mimicry target	Immune cross-reactivity with endothelial HSP60 → autoimmune-like injury	Serum; tissue	ELISA; immunohistochemistry	[45]
Nitric oxide bioavailability (eNOS pathway surrogates)	Endothelial function markers	Reduced NO signaling links inflammation/oxidative stress to dysfunction	Plasma (nitrates/nitrites)	Griess assay; chemiluminescence (research)	[34]

Local biomarker elevation does not necessarily translate linearly into systemic cardiovascular risk; rather, these markers primarily reflect periodontal inflammatory burden and may contribute indirectly to systemic risk assessment when interpreted alongside circulating biomarkers and clinical variables

### Inflammatory cytokines

**Fig. 1:**
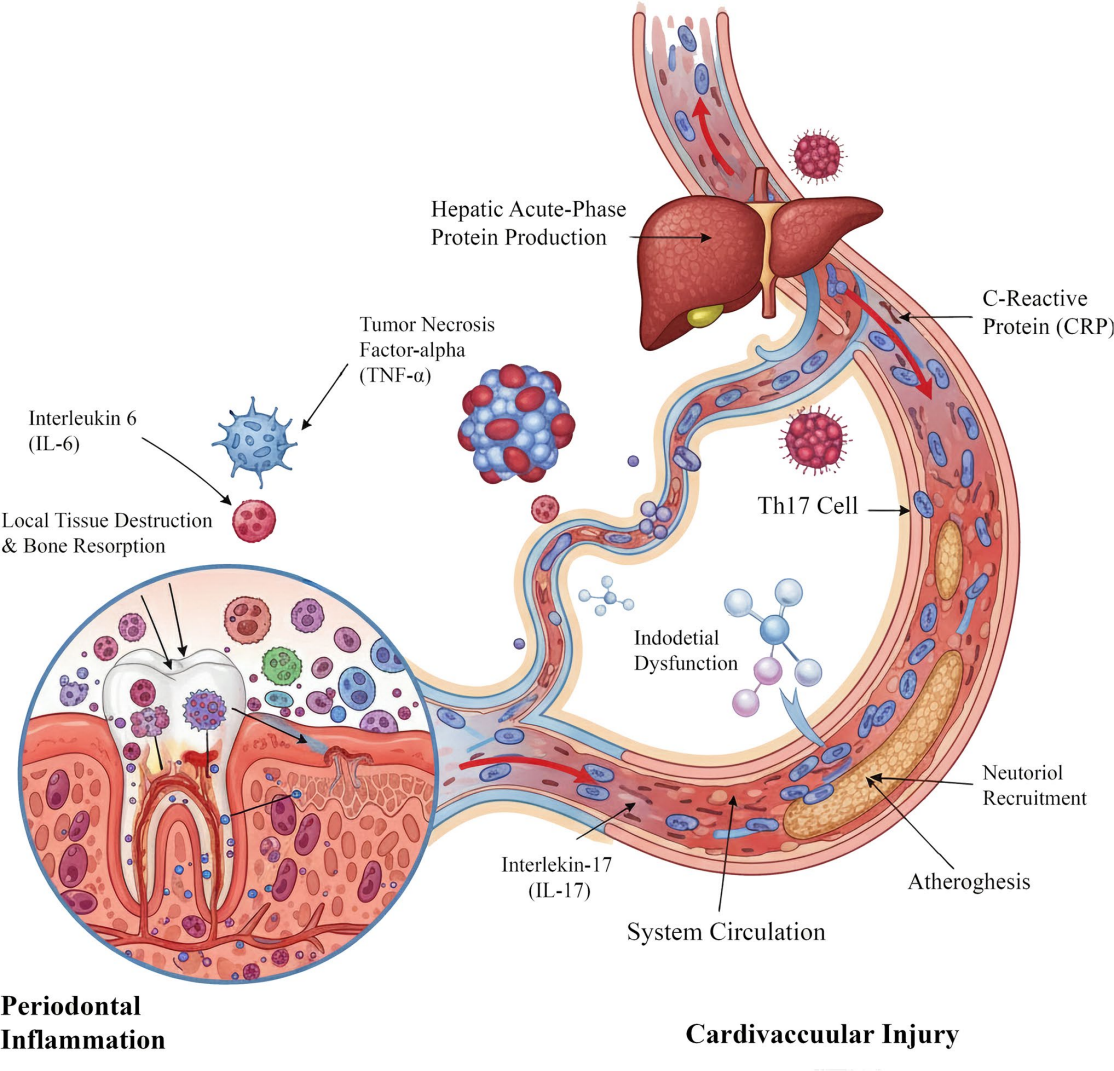
Schematic representation of elevated inflammatory cytokines linking periodontal disease to cardiovascular injury. Periodontal infection stimulates local release of IL-6, TNF-α, and IL-17, driving tissue destruction and bone resorption. These cytokines enter systemic circulation, induce hepatic acute-phase protein production (e.g., CRP), promote neutrophil recruitment, and contribute to endothelial dysfunction and atherogenesis

Inflammatory cytokines are an important class of biomarkers that link periodontal disease to cardiovascular disease, such as IL-1β, IL-6, TNF-α, and interleukin-17 (IL-17), which are elevated in periodontitis and contribute to systemic inflammatory burden [46]. IL-1β and TNF-α are mediators of local tissue destruction and bone resorption and promote the release of systemic cytokines [1, 34]. Particularly, IL-6 is an important mediator of hepatic acute-phase protein production, such as CRP, which is closely linked to cardiovascular disease risk [34]. IL-17, secreted by T helper (Th)17 cells, augments recruitment of neutrophils and enhances vascular inflammation, thereby linking it to endothelial dysfunction and atherogenesis [47]. Cytokines are elevated in periodontal patients, such as those indicated above; therefore, they can be seen as potential mechanistic biomarkers that link periodontal inflammation to endothelial injury and cardiovascular disease [2]. As illustrated in Fig. 1, periodontal infection results in locally induced cytokine release, which is then disseminated systemically, leading to hepatic acute phase responses and ultimately vascular dysfunction and atherogenesis.

### Acute-phase proteins

Acute-phase proteins are systemic markers of inflammation that increase in response to periodontal infection and contribute to cardiovascular pathology. C-reactive protein is the most studied and is consistently found to be increased in patients with periodontitis. Elevated hepatic production of C-reactive protein occurs in response to systemic inflammation, driven by pro-inflammatory cytokines such as interleukin-6 (IL-6) [18]. C-reactive protein shows increased levels associated with a more prevalent degree of endothelium dysfunction; instability of atheromatous plaque; and increased cardiovascular risk [48]. Fibrinogen, a second acute-phase reactant, enhances platelet aggregability and subsequent indirect and direct effects on blood clotting mechanisms, which produce the pro-inflammatory state noted in both periodontitis and cardiovascular disease [26]. Serum amyloid A (SAA) has also again demonstrated to be found in the serum of patients with increased periodontal disease. It has been linked to vascular inflammation and the subsequent alteration of tissue metabolism, which promotes atherogenesis [49]. These proteins, in sum, demonstrate a membership not only as markers of systemic inflammation but also as reliable predictors of cardiovascular events, which emphasizes their position as common biomarkers in the periodontal-cardiovascular axis [2].

### Matrix metalloproteinases (MMP-8/aMMP-8)

In addition to its role as a biomarker of periodontal tissue destruction, MMP-8 has also been associated with systemic inflammatory processes linked to cardiovascular diseases. Elevated levels of MMP-8 have been detected in patients with acute coronary syndromes and atherosclerotic plaques, suggesting that collagen degradation and matrix remodeling contribute to vascular instability and plaque progression. Importantly, the aMMP-8 reflects ongoing enzymatic activity and therefore provides a more accurate indicator of active periodontal inflammation compared with total MMP-8, which includes both inactive precursor and active forms. Consequently, aMMP-8 has emerged as a clinically relevant biomarker for detecting active periodontal tissue destruction and monitoring treatment response, particularly when measured in oral fluids such as gingival crevicular fluid, saliva, or mouthrinse samples [27–29, 50–53].

### Oxidative stress markers

Oxidative stress plays an integral role in the association between periodontal disease and cardiovascular disease, and various biomarkers are used to assess vascular injury. Myeloperoxidase (MPO), released from activated neutrophils, produces reactive oxidants that lead to low-density lipoprotein (LDL) oxidation, endothelial dysfunction, and atherosclerotic plaque formation [35]. Increased levels of MPO may also be associated with plaque destabilization and an increased risk of acute coronary events [36].

Malondialdehyde (MDA), a product of lipid peroxidation, measures oxidative damage to cellular membranes and correlates with periodontal inflammation and atherogenesis [37]. 8-OHdG is a measure of oxidative DNA damage and is increased in periodontitis patients and associated with vascular oxidative stress, contributing to endothelial injury and plaque vulnerability [39]. Altogether, these oxidative stress biomarkers support the findings of the contribution of oxidative stress to both plaque formation and instability, demonstrating their importance in the periodontal–cardiovascular axis [38].

### Lipid metabolism and adhesion molecules

The metabolic alterations of lipids and endothelial adhesion molecules represent important pathways linking pathological states, which are responsible for the alteration of the dyslipidemic state, infection, and inflammation to the vascular wall. Lipoprotein-associated phospholipase A2 (Lp-PLA2) a member of the family of enzymes associated with low-density lipoprotein (LDL) cholesterol, hydrolyzes oxidized phospholipids, giving rise to proinflammatory lysophosphatidylcholine (LPC) and oxidized fatty acids, seen in atherogenic precipitation of atherosclerotic plaques [28]. Increased activity of Lp-PLA2 has been noted in those with periodontitis and have been correlated with increased cardiovascular disease risk, as indicated by the investigator [18].

ICAM-1 and VCAM-1 are upregulated in response to periodontal pathogens and systemic inflammatory cytokines and facilitate the adhesion of white blood cells and their transmigration through the vascular endothelium [31, 41]. This creates a state of endothelial dysfunction and promotes the development of inflamed, unstable plaques. Lp-PLA2, ICAM-1, and VCAM-1 represent mechanistic linkages between microbial dysbiosis, lipid dysregulation, and vascular inflammation, providing a link between the two endpoints and demonstrating their potential utility as biomarkers and therapeutic targets both in periodontal disease and cardiovascular disease [2].

### Microbial and immune-related biomarkers

Microbial and immune-related biomarkers provide direct evidence of the link between periodontal pathogens and cardiovascular disease. Antibodies to Porphyromonas gingivalis are frequently elevated in patients with periodontitis and have been associated with increased risk of cardiovascular disease due to both microbial exposure and host immune response [42]. Lipopolysaccharides (LPS) of Gram-negative periodontal bacteria enter the systemic circulation during transitory bacteremia and promote activation of Toll-like receptors, which trigger systemic inflammation and endothelial dysfunction [43]. Furthermore, bacterial DNA (including P. gingivalis) can be found within human atherosclerotic plaques, which clearly implicates direct microbial involvement in the development of the plaque [14, 30]. Through molecular mimicry and immune cross-reaction, vascular injury is produced since the heat shock proteins of the bacteria share homology with such human proteins, which produces endothelial damage characteristic of autoimmunity [44]. Taken together, these microbial and immune-related biomarkers provide further biological plausibility that periodontal pathogens play a role in atherogenesis and cardiovascular disease and underscore the importance of information regarding markers of oral infection in systemic disease risk assessment.

## Mechanisms linking periodontal health to cardiovascular disease

**Fig. 2:**
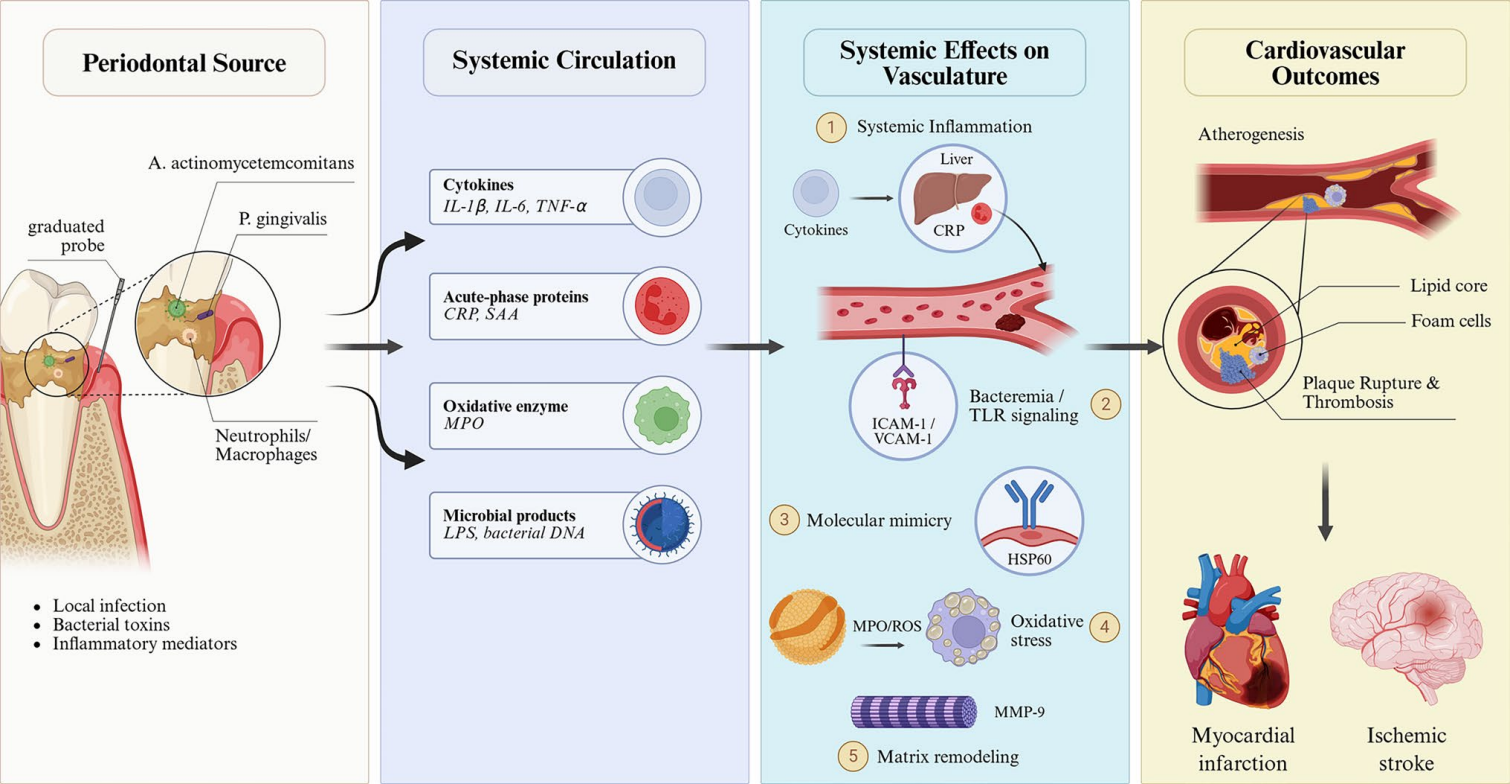
Periodontal infection triggers systemic inflammation, bacteremia, oxidative stress, and immune cross-reactivity, promoting endothelial dysfunction, atherogenesis, and cardiovascular events

The biological plausibility linking periodontal disease to CVD rests on several interrelated mechanisms that converge on systemic inflammation, endothelial dysfunction, and atherogenesis. As illustrated in Fig. 2, periodontal pathogens and host immune responses generate inflammatory mediators, microbial by-products, and oxidative stress that enter systemic circulation, driving endothelial dysfunction, atherogenesis, and ultimately cardiovascular events.

### Systemic inflammation

Localized immune responses are activated by periodontal pathogens (*P. gingivalis* and *Aggregatibacter actinomycetemcomitans*) which induce the continuous release of inflammatory cytokines (IL-1, IL-6, and TNF-α) and chemokines to the gingival tissues (Fig. 2). These inflammatory mediators are released into circulation, causing a systemic inflammatory response and hepatic production of acute-phase proteins such as CRP and serum amyloid A. There is a significant correlation between systemic inflammation and increased levels of CRP, which indicates cardiovascular events. CRP may be used as a marker of systemic inflammation or to exhibit the participation of the inflammatory changes in the process of vascular pathology [1, 33].

### Bacteremia and endothelial dysfunction

Common oral activities, such as chewing and tooth brushing, can force periodontal pathogens and their virulence factors, such as LPS and gingipains, into the bloodstream. Once in the system, these bacteria attach to and invade vascular endothelial cells, with the subsequent activation of adhesion molecules (ICAM-1 and VCAM-1) (Fig. 2) and recruitment of leukocytes. Activation of these endothelial cells leads to disruption of nitric oxide (NO) signaling, vasodilation, and formation of a pro-atherogenic environment suitable for lipid deposition and plaque formation [14, 34].

### Immune cross-reactivity and molecular mimicry

The structural similarities of the bacterial heat-shock proteins (HSP60) and the host HSPs elicit an autoimmune-like response. Antibodies made against the bacterial HSPs cross-react with the endothelial HSPs in the host, which leads to immune-mediated injury to the blood vessels. This phenomenon of molecular mimicry (Fig. 2) also leads to chronic vascular inflammation, smooth muscle proliferation, and accelerated plaque formation [44].

### Oxidative stress and lipid peroxidation

The inflammation in the periodontium of periodontal disease attracts neutrophils and macrophages that secrete MPO and ROS (Fig. 2). These oxidative intermediates promote the oxidation of low-density lipoprotein (LDL) and lead to oxidized LDL, a potent stimulus for the production of foam cells and atherogenesis. The corresponding levels of MDA and 8-OHdG represent the oxidative injury to the lipid fraction and the DNA, respectively, and relate periodontal oxidative stress to the vascular and plaque rupture susceptibility [35, 38].

### Matrix metalloproteinases and plaque destabilization

Pathogens and cytokines involved in periodontal disease stimulate the synthesis of matrix metalloproteinases (MMP-2, MMP-8, MMP-9), enzymes that degrade components of the extracellular matrix. Increased MMP activity within atherosclerotic lesions weakens the fibrous caps of the lesions, thus predisposing the plaques to rupture. The rupture of a plaque leads to exposure of the thrombogenic material in the plaque to the blood, leading to platelet aggregation and to the formation of thrombi, the immediate causes of acute myocardial infarction and ischemic strokes [45].

### Integrated pathophysiology

Taken together, these mechanisms underscore a multi-factorial scheme in which periodontal infections act both as chronic systemic inflammatory stimuli and as direct microbial challenges to the vascular endothelium. The interplay of inflammation driven by cytokines, oxidative stress, immune mimicry, and degradation of the extracellular matrix leads to biological pathways linking oral disease with cardiovascular pathology [8, 16, 34].

## Evidence from clinical and experimental studies

### Epidemiological studies

An extensive body of epidemiological literature strongly links periodontitis with an increase in the incidence of CVD. Many large prospective investigations have also documented an increased risk of developing coronary heart disease (CHD), stroke, and CVD mortality in patients who have poor periodontal health, independent of traditional risk factor considerations. For instance, the Health Professionals Follow-up Study by Joshipura et al. examined over 44,000 men and found that participants in the study with poor dental health had a highly significant increase in the risk of CHD even after controlling for diabetes mellitus, cigarette smoking, and other confounding factors [32]. Similarly, the ARIC (Atherosclerosis Risk in Communities) study demonstrated that advanced periodontitis with increased probing depth and clinical attachment loss was associated with a highly significant increase in CVD mortality, independent of the usual risk determinants [54].

Meta-analytic data also support these findings. Bahekar et al. reported on 15 studies with a meta-analysis of 5 prospective cohorts and noted that periodontitis increased the risk of CHD by 14% (RR = 1.14; 95% CI 1.07–1.21; *P* < 0.001) [9]. Importantly, several studies provide evidence for a dose–response relationship whereby increasing severity of periodontitis (as assessed clinically by probing depth, clinical attachment loss, and tooth loss) is associated with increased cardiovascular morbidity. In a cross-sectional study by Holmlund and colleagues (2006), it was seen that patients with fewer than 10 remaining teeth had a higher prevalence of myocardial infarction and hypertension compared to patients with retained 25 teeth or more, independent of other cardiovascular risk factors [55].

This dose–response relationship has been confirmed by a prospective cohort meta-analysis. Cheng et al. (2018) examined nearly 880,000 people studied in 17 cohorts and determined that each incremental dose of 2 teeth lost was associated with a 3% increase in risk of CHD and stroke (RR per 2 teeth lost = 1.03; 95% CI 1.01–1.04), thus supporting a linear relationship between tooth loss and cardiovascular morbidity [56]. Furthermore, longitudinal findings expand this field beyond the first sick to subsequent illnesses. An important cohort analysis extending over a period of 10 years in patients with already established CVD revealed that advanced periodontitis was significantly associated with an increase in cardiovascular morbidity (adjusted HR = 1.26; 95% CI 1.00–1.58), suggesting that the status of oral health may well relate to the results of secondary prevention of cardiovascular illness [56].

Collectively, these findings provide strong population-level evidence that periodontitis is not only a marker of oral health decline but also a contributor to systemic vascular risk, highlighting the need to integrate periodontal status into cardiovascular risk assessment.

### Interventional studies

Clinical intervention studies provide evidence that periodontal therapy may be beneficial in the treatment of systemic inflammation and vascular function. Non-surgical periodontal therapy consisting primarily of scaling and root planning with or without oral hygiene instruction, combined with adjunctive antimicrobial or anti-inflammatory therapy, has been shown to have a significant and worthwhile effect on the various circulating biomarkers, including CRP and IL-6, representing the decreased body burden of systemic inflammation [18, 57]. More intensive treatment modalities may also embrace periodontal surgical treatment, systemic antibiotics (amoxicillin and metronidazole, for example), or molar extractions for extensive disease. These various therapeutic modalities are attitudes designed to eradicate or dramatically reduce the microbial load of the diseases, eliminate local inflammatory reaction, and consequently eliminate systemic inflammatory exudation, which may explain the increased vascular function.

In a randomized controlled trial of patients with advanced periodontal disease, Tonetti et al. [17] reported that patients received either intensive periodontal therapy (including scaling and root planning, extractions if required, and local antibiotic therapy) or community-based standard care [17]. The intensive treatment resulted in significant improvements in endothelial function, as assessed by measurement of endothelium-dependent vasodilation (FMD) of the brachial artery. Although an acute systemic inflammatory response was apparent as early as 24 h after treatment (transient increases in CRP and IL-6), the long-term results were promising. At six months, the intensive treatment group had a significant absolute increase in FMD of 2.0% (*P* < 0.001) and reductions in soluble E-selectin levels, seen in the improved responsiveness of the endothelium.

Seinost et al. [58] likewise found that endothelial function was significantly impaired in periodontal patients compared to healthy controls at baseline (FMD: 6.1% vs. 8.5%; *P* = 0.002). With intensive periodontal therapy, FMD improved to 9.8% in 3 months (*P* = 0.003), as well as a significant reduction in CRP levels (from 1.1 to 0.8 mg/L; *P* = 0.026), further supporting the vascular benefit of treatment [58].

In a pilot trial, Elter et al. [59] followed 22 adults with moderate to severe periodontitis who had undergone comprehensive periodontal therapy (periodontal scaling and root planning, oral hygiene reinforcement, and restraining as required). After follow-up at 1 month, improvement was noted in FMD and a reduction in IL-6, with a trend noted for decreased CRP, demonstrating the short-term systemic effects of periodontal therapy [59].

Taken together, these interventional studies imply that periodontal treatment has the ability to partially reverse early vascular dysfunction and lower systemic inflammation. Nonetheless, the present evidence base is characterized by small sample sizes, indirect measures, and relatively short follow-up periods. There is a necessity for larger and better-designed randomized controlled trials to determine whether vascular and inflammatory improvements are mediated due to results' causative effects on reductions in cardiovascular morbidity and mortality. A summary of the key clinical trials, including compositions of study populations, types of therapies given, follow-up periods, and main outcomes, is shown in Table 2.

**Table 2:** Summary of key interventional studies evaluating periodontal therapy and vascular outcomes

Study	Population	Therapy	Follow-up	Key outcomes
Tonetti et al. 2007 (NEJM) [17]	120 patients with severe periodontitis	Intensive periodontal therapy: SRP, extractions if indicated, systemic antibiotics (amoxicillin + metronidazole), oral hygiene instruction	6 months	↑ FMD (absolute difference 2.0%; *P* < 0.001); ↓ soluble E-selectin; transient ↑ CRP and IL-6 at 24 h but long-term ↓ systemic inflammation
Seinost et al. 2005 (Am Heart J) [58]	30 patients with severe periodontitis vs 31 healthy controls	Non-surgical periodontal therapy (SRP + oral hygiene reinforcement)	3 months	Baseline impaired FMD (6.1% vs 8.5% controls); post-therapy ↑ to 9.8% (*P* = 0.003); ↓ CRP (1.1 → 0.8 mg/L; *P* = 0.026)
Elter et al. 2006 (Am Heart J) [59]	22 adults with moderate-to-severe periodontitis	Comprehensive periodontal therapy (SRP, oral hygiene reinforcement, localized treatment as required)	1 month	↑ FMD; ↓ IL-6; trend toward ↓ CRP levels

### Experimental and animal studies

Evidence for the relationship between periodontal pathogens and atherogenesis has been obtained from experimental models and animal studies. In vivo studies have shown that intravenous injection of oral bacteria, such as *P. gingivalis*, induces an acceleration of atheromatous lesions and systemic inflammation, and arterial lipid deposition [60, 61].

Li et al. [60] tested the effect on ApoE-deficient mice, the accepted model of atherosclerosis, of repeated systemic doses of *P. gingivalis*, a major pathogen of periodontitis. Mice receiving intravenous injections of *P. gingivalis* over 10 to 24 weeks exhibited significantly increased aortic atherosclerotic lesion area compared to controls, with a twofold increase in plaque burden in both histological and in face analyses. Noteworthy was the finding of bacterial DNA in the aortic tissues, and systemic mediators of inflammation, such as IL-6 and TNF-α were also elevated, showing a further systemic proatherogenic effect of such infected mice.

Gibson et al. [61] recommended studies of the entities involved in innate immune responses to *P. gingivalis* and how they were mediated to important vascular pathology. In TLR-2 receptor mutant mice, unable to recognize gram-negative bacteria due to their lipoproteins, there was a significant decrease in atherogenesis due to the *P. gingivalis* challenge. This again indicated that it was in the TLR receptor pathways that important mediators were involved in the inflammation-induced injury to the vascular wall, due to periodontal pathogens.

Extending these insights, Velsko et al. [62] showed that oral infection of ApoE-null mice with *P. gingivalis* resulted not only in inflammatory events such as alveolar bone loss and elevation of serum inflammatory mediators (e.g., serum amyloid A, oxidized LDL), but also direct invasion of *P. gingivalis* into the aorta. Viable *P. gingivalis* was detected in oral tissues and in the vascular endothelium through the use of in situ fluorescent hybridization, and plaque burden was found to be significantly greater in infected mice (24 weeks) compared to uninfected controls (*P* < 0.01), thus confirming a causative relation between chronic oral infection and the progression of atherosclerosis.

Importantly, this experimental evidence is supported by human study findings. Kozarov et al. detected viable *P. gingivalis* and *A. actinomycetemcomitans* in human atherosclerotic plaques (2005) [30] suggesting direct translocation of the microbiota from oral cavity to vascular tissues. The invasion of bacteria was established by culture and molecular techniques, indicating that presence was not merely passive, but that three bacteria were established in the arterial wall.

Armingohar et al. [63] provided molecular evidence that vascular lesions in patients with chronic periodontitis harbored greater diversity and load of bacterial DNA, including *P. gingivalis*, than those individuals not suffering from periodontitis. The polymicrobial DNA signatures in aortic and carotid specimens were elucidated, using 16S rDNA sequencing, and scanning electron microscopy confirmed the presence of bacterial structures. While only one of the biopsies confirmed *P. gingivalis* by species-specific polymerase chain reaction, all vascular samples held culturable or unculturable microbial sequences, indicating the polymicrobial nature of systemic dispersion.

Multiple groups have detected periodontal pathogens within human atheromas using complementary techniques, including the use of species-specific polymerase chain reaction, broad-spectrum utilization of 16S rRNA polymerase chain reaction coupled with sequencing, and, in some studies, culture of viable organisms and/or in situ localization. In a classic carotid endarterectomy series, Haraszthy et al. examined 50 plaques and found DNA from at least one periodontal pathogen in 44% of specimens; detection frequencies included *P. gingivalis* (~ 26%), *A.actinomycetemcomitans* (~ 18%), *Tannerella forsythia* (~ 30%) and *Prevotella intermedia* (~ 14%) supporting direct microbial translocation into vascular lesions [64]. Extending beyond DNA detection, Kozarov et al. reported viable, invasive *A. actinomycetemcomitans* and *P. gingivalis* recovered from human atherosclerotic plaques, demonstrating bacterial persistence within vascular tissue rather than surface contamination [30]. Mechanistic reviews and tissue studies have additionally documented the localization of oral bacterial signatures along with host response markers within carotid and coronary lesions and have led to the outlining of a biologically plausible etiology for periodontal pathogens to contribute to vascular inflammation and atherogenesis [14]. Thus, the findings from these different lines of evidence converge to provide epidemiological and interventional data by outlining the possible etiological pathways from oral infection into vascular disease.

Expanded animal models beyond mice, including rats, rabbits, ferrets, and nonhuman primates, have been used to simulate periodontitis and systemic effects. Each model has strengths and limitations regarding immunological fidelity and cost. Ligature-induced periodontitis remains the most common model for inflammation and alveolar bone loss. Humanized bacterial models, in which mice are colonized with human-derived oral flora, may provide greater translational accuracy [65, 66].

Experimental periodontitis has also been shown to increase systemic oxidative stress, measured through markers such as total oxidant status (TOS) and lipid peroxidation [67]. Coexisting disease models (e.g., combining periodontitis with kidney disease) [68] have demonstrated synergistic systemic damage. Furthermore, spatial omics and organ-on-chip models emerge to localize bacteria and metabolites within vascular tissue, supporting translational insights.

Collectively, the findings from epidemiological, interventional, and experimental studies provide converging evidence for a biologically plausible association between periodontitis and CVD. Large cohort studies have established a consistent association and dose–response relationship between periodontal severity and cardiovascular outcomes [9, 56, 69, 70]. Interventional trials have shown that periodontal therapy can reduce the systemic inflammatory markers and will also improve endothelial function, thus giving evidence for the possible reversibility of cardiovascular risk [17, 57–59]. Biological plausibility has further been confirmed by experimental and animal studies, which show that oral pathogens, mainly Porphyromonas gingivalis, will accelerate atherogenesis and are detectable within the vascular plaques [14, 30, 60].

The consistent detection of bacterial DNA in the atheromatous tissues, together with the evidence on progression of lesions in infected animals, lends support to the model in which chronic oral infection plays the part of a persistent inflammatory trigger for vascular pathology. Together, these complementary lines of evidence serve to highlight periodontitis as a remediable risk factor for CVD and thus underline the importance of integrated oral and systemic health strategies in clinical practice and research.

## Diagnostic and prognostic potential

Periodontal biomarkers are a promising tool for improving the diagnosis and prognosis of CVD. Among these, CRP, IL-6, and MPO appear to have the most consistent correlation with systemic inflammation and cardiovascular risk. In regard to CRP, the elevation of CRP in patients who have periodontitis serves not only to reflect the hepatic acute phase responses, but also to predict the incidence of myocardial infarction and stroke, thus making CRP an excellent agent to be included in the stratification of risk for CVD [18, 48].

IL-6 is a primary upstream cytokine that stimulates production of CRP and, similarly to CRP, is associated with progression of atherosclerosis and adverse outcomes [71]. MPO is an oxidative enzyme released from activated neutrophils and is involved in the process of oxidation of low-density lipoproteins (LDL) as well as plaque instability, and data support that increased circulating MPO correlates with acute coronary syndrome [35, 36].

With the recent advances in the techniques from which these diagnostic tools can be evaluated, these biomarkers have the potential to be evaluated not only in serum but also in saliva and gingival crevicular fluid (GCF). These markers reflect not only local periodontal inflammation but also the systemic inflammatory burden [72]. The evolution of POC diagnostic platforms such as lateral flow assays and biosensing platforms opens the way for these biomarkers (CRP, IL-6, MPO) to be evaluated rapidly and chair-side from oral fluids and/or capillary blood [73]. Such methods may serve to accurately measure elevation in cardiovascular risk in a population in the dental and medical clinical settings and help to bridge the gap between oral and systemic health.

A systematic review of salivary biomarkers and CVD concluded that among ~ 40 candidate markers, salivary CRP demonstrated the most consistent positive associations, whereas results for other markers (e.g. MMP-9, troponin I, MPO) were more heterogeneous [74]. In patients with periodontitis with or without acute myocardial infarction (AMI), salivary CRP and MIP-1α levels were significantly elevated, and adiponectin was reduced, supporting the potential use of salivary inflammatory biomarkers in cardiovascular risk screening [75]. Yet, the heterogeneity of sample collection timing, salivary flow, dilution, and assay sensitivity continues to complicate cross-study comparability, suggesting the need for strict standardization [74].

Not all biomarkers discussed in this review have equal value for cardiovascular risk assessment. Circulating markers such as hs-CRP, IL-6, fibrinogen, and MPO have stronger evidence as systemic indicators associated with cardiovascular risk, although they remain nonspecific and may rise in many inflammatory states. By contrast, oral-fluid biomarkers such as aMMP-8, IL-1β, and MMP-9 are more informative for periodontal disease activity than for direct cardiovascular risk prediction. Similarly, antibodies to Porphyromonas gingivalis may reflect microbial exposure and possible biological linkage to vascular disease, but they do not by themselves establish active periodontal disease or current cardiovascular instability. For this reason, periodontal biomarkers are best viewed as complementary tools that may refine risk phenotyping rather than as stand-alone cardiovascular prognostic markers.

**Fig. 3:**
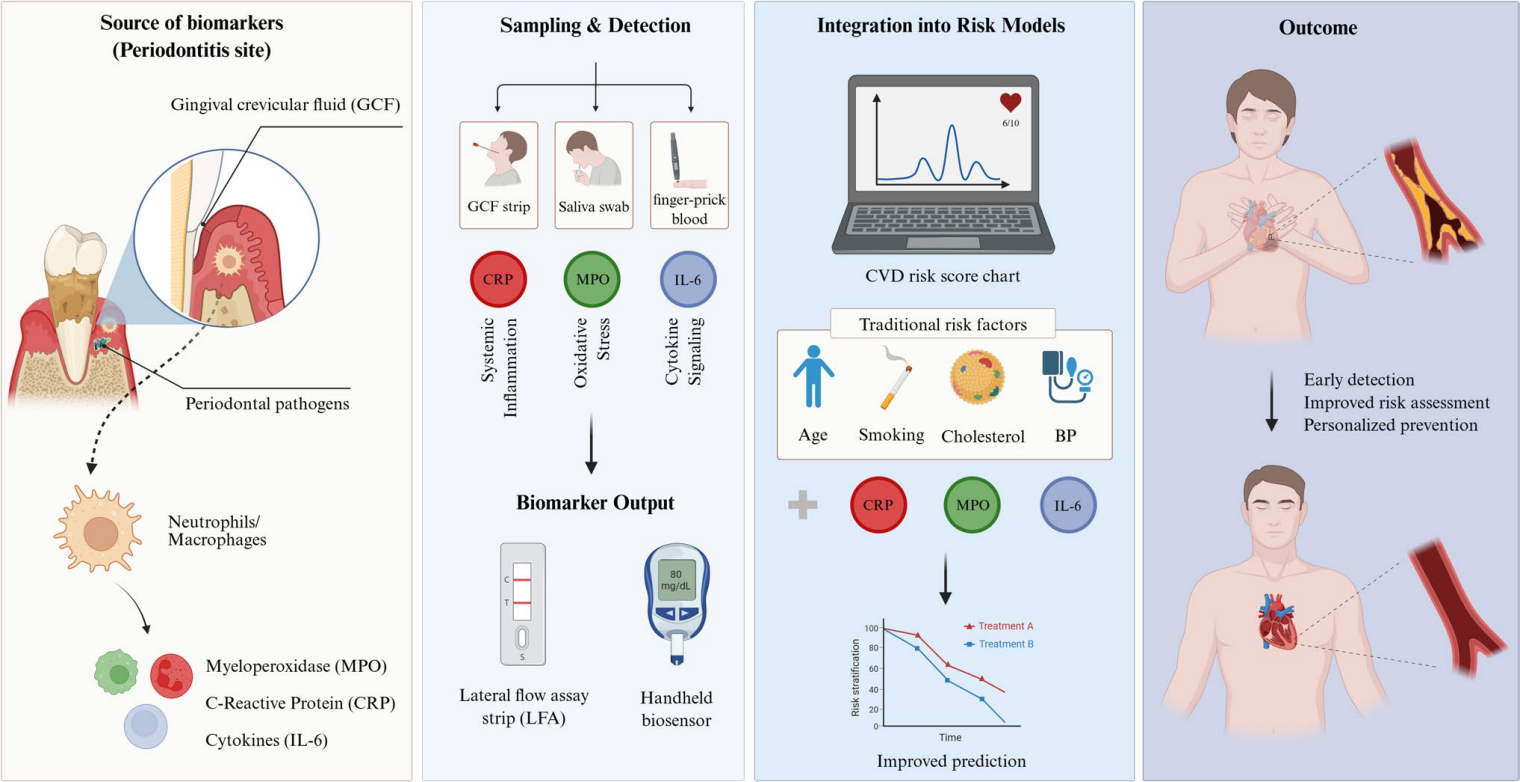
Periodontal biomarkers (CRP, IL-6, MPO) detected in saliva, GCF, and blood integrated into POC tools to improve cardiovascular risk assessment

As demonstrated in Fig. 3, biomarkers released from inflamed periodontal tissues gain access to saliva, GCF, and blood, where they can be measured by POC devices. The significance of these findings is that the incorporation of these measurements into cardiovascular risk algorithms will enhance the precision of predicting adverse events, allow for early diagnosis, and lead to the formulation of more individualized preventive strategies.

The incorporation of periodontal biomarkers into established CVD risk estimates, such as those derived from the Framingham Risk Score or pooled cohort equations, would enhance predictive value, particularly among individuals at intermediate risk, where current estimates for 10-year CVD events or other serious complications are shown to be deficient [2]. An integration of traditional cardiovascular risk factors along with knowledge of biomarkers from oral diagnostic tests may permit a precision medicine model directing tailored outcomes and interdisciplinary management. Longitudinal studies and clinical trials will be necessary in the future to determine the validity of biomarker cut-off thresholds, standardization of methods for sample collections, and to assess the incremental predictive value of these markers in large, diverse populations.

Metabolomics has identified disease-associated small molecules, such as amino acid derivatives, short-chain fatty acids, and lipid mediators. A recent metabolomics systematic review found 56 metabolites recurrently associated with periodontitis; many relate to amino acid and organic acid metabolism [76]. There is increasing recognition that periodontal disease and atherosclerotic disease may share overlapping metabolic perturbations, such as lipid mediator dysregulation, oxidative stress metabolites, and microbe-derived short-chain fatty acids [77]. Proteomics and microbiome profiling also reveal distinct signatures linked to periodontitis and vascular inflammation [78]. Multi-omic approaches integrated with imaging and clinical data via artificial intelligence (AI) could yield comprehensive risk models.

Standardization of biomarker thresholds, biological validation, and cost–benefit evaluations remain barriers to routine use. Longitudinal studies must assess biomarker variation and stability across populations and disease states before clinical integration can proceed.

Machine learning and AI models have been employed to correlate oral imaging biomarkers (e.g. intraoral fluorescence) and periodontal indices with systemic health outcomes, offering an integrative pathway to noninvasive screening [79]. Multimodal risk models combining omics, clinical, imaging, and biomarker data may allow data-driven risk phenotyping rather than additive biomarker approaches.

## Challenges and limitations

Despite promising evidence, several challenges limit the translation of periodontal biomarkers into cardiovascular risk assessment. Many existing studies are cross-sectional or short-term; the stability of biomarker signatures over time (intra-individual variation) is underexplored. Biological variability (e.g., circadian rhythms, diet, saliva flow, comorbidities) may confound the interpretation of oral fluid biomarkers. The incremental predictive gain of periodontal biomarkers beyond existing cardiovascular risk markers is still unproven.

### Variability in biomarker measurement methods

There is considerable variability in biomarker sampling and measurement methods, including sample site (saliva, gingival crevicular fluid, serum), sensitivity of assays performed, and absence of standardized thresholds. This variability presents a challenge in comparing studies and the application of clinical effects [72]. Standardization of sampling techniques and validated cut-off values will be important in advancing the usefulness of biomarkers. For salivary and oral-fluid point-of-care devices, standardization of sampling procedures is especially critical. Factors such as time of day, fasting status, recent oral hygiene practices, eating or drinking, stimulated versus unstimulated saliva, and variable salivary flow rates may substantially affect analyte concentration through dilution or concentration effects. Without harmonized pre-analytical sampling protocols, salivary biomarker measurements may show poor reproducibility and may generate misleading or false results.

### Confounding factors

Lifestyle habits and systemic factors, including cigarette smoking, diabetes, and obesity, influence systemic inflammation and cardiovascular outcomes independently, thus complicating the isolation of the specific contribution of periodontal disease [11, 80]. Furthermore, improvements in systemic biomarkers, which occur in some interventional studies and include the lowering of CRP, which is observed with periodontal therapy, may be partly a reflection of the influence on non-specific infections or improved oral hygiene, rather than a direct periodontal-dependent cardiovascular effect. These variables require rigorous assessment and consistent reporting to enable causal inference if strengthened. In epidemiological studies, the independent contribution of periodontitis to cardiovascular outcomes is typically examined using multivariable regression models that adjust for shared risk factors such as age, sex, smoking, diabetes, obesity, hypertension, and socioeconomic status. Some studies additionally apply matching, stratified analyses, or sensitivity analyses to reduce imbalance between exposed and unexposed groups. However, because periodontitis and cardiovascular disease share multiple behavioral and metabolic determinants, residual confounding and reverse causation remain difficult to exclude fully. Accordingly, even when associations persist after adjustment, these findings should be interpreted as supportive rather than definitive evidence of causality, particularly in the absence of large long-term randomized trials with hard cardiovascular endpoints.

### Limited longitudinal and interventional evidence

As observational studies continue to identify associations, evidence from longitudinal and interventional studies is limited. Most clinical trial data are focused on short-term changes in systemic markers after periodontal therapy, and the number of large randomized controlled trials associating periodontal treatment with hard cardiovascular end points, such as myocardial infarction or stroke [2, 17] is quite limited. Studies carefully designed and well-powered to be long-term are needed to determine the clinical impact of periodontal therapy on cardiovascular outcomes.

### Validity of conclusions

Although biological plausibility and observational evidence support an association between periodontitis and cardiovascular outcomes, definitive evidence that periodontal treatment reduces hard cardiovascular endpoints such as myocardial infarction, stroke, or cardiovascular mortality is still lacking. Most of the evidence at present is associative and mechanistic rather than definitive. Intervention studies using periodontal therapy, such as the NEJM trial by Tonetti et al. [17] have shown short-term improvements in markers of systemic inflammation (e.g., CRP) and vascular function (flow-mediated dilation), but these studies are small of short duration and use surrogate endpoints [17, 81].

At present, there is no definitive evidence from large-scale randomized controlled trials that treatment of periodontal disease reduces the incidence of hard cardiovascular outcomes such as myocardial infarction or stroke. Accordingly, caution should be exercised in drawing conclusions about the causal role of periodontitis in CVD. At present, findings indicate that periodontal therapy is likely to have short-term systemic benefits, but that well-powered long-term randomized controlled trials (RCTs) are necessary to determine if this translates into decreased cardiovascular events [2, 17, 81].

Taken together, periodontal biomarkers, especially when extended via omics and integrated into modeling frameworks, hold promise for bridging oral-systemic assessment. But before broad clinical adoption, rigorous validation, large prospective cohorts, and assay standardization are imperative. Additional considerations include the need for harmonized definitions of periodontitis severity, improved adjustment for comorbidities in statistical models, and multi-biomarker panels that integrate microbial, inflammatory, and metabolic data. Future trials should be designed with composite endpoints and real-world populations to improve external validity.

## Future directions

To strengthen the clinical translation of periodontal biomarkers in CVD risk assessment, several future directions are critical.

### Emerging omics biomarkers and AI integration

Along with well-established inflammatory, oxidative, and microbial markers, the field is expanding with the availability of omics approaches. Proteomics has allowed the discovery of new inflammatory mediators and matrix-degrading enzymes, and metabolomics has revealed lipid mediators and oxidative stress metabolites that are shared by periodontitis and atherosclerosis [22, 23]. Microbiome profiling has revealed distinct bacterial signatures associated with systemic inflammation and vascular risk [14, 30]. In addition to proteomics and metabolomics, lipidomics, glycomics, and transcriptomics (local and systemic) may uncover novel mediators linking periodontitis and atherosclerosis. Microbiome profiling has revealed distinct bacterial signatures associated with systemic inflammation and vascular risk.

Spatial omics (e.g., imaging mass spectrometry, spatial transcriptomics) could localize microbial metabolites or host responses directly within vascular lesions, correlating microbial presence with lesion phenotype. Integration of microbial metabolite signatures (e.g., volatile organic compounds, short-chain fatty acids, LPS derivatives) with host biomarkers can better capture the functional host-microbe interface. Multi-cohort consortia with harmonized sampling (e.g., the International Periodontal-Cardiovascular Consortium) could accelerate validation of omics signatures.

Furthermore, AI and machine learning offer opportunities to integrate omics data, clinical biomarkers, and imaging to enhance precision risk stratification [82]. Future research should focus on validating omics-derived signatures in large cohorts and developing clinically applicable AI-driven models.

Newer platforms, such as spatial transcriptomics and single-cell proteomics, may provide insight into the tissue localization of inflammatory and microbial signals in atherosclerotic plaques. Integration with imaging modalities (e.g., PET/CT) could enable functional–anatomical correlations for high-risk plaque phenotyping.

### Refined animal and in vitro models

Development of humanized vascular/oral microbiome models, e.g., mice colonized with human oral microbial communities plus humanized immune‐vessel grafts, could better simulate human pathophysiology. Use of organ-on-chip systems combining periodontal tissue, endothelial barrier, and flow conditions may allow dynamic modeling of bacterial translocation and inflammatory crosstalk. Dual disease models (e.g., combining periodontitis with hypertension, diabetes, hyperlipidemia) can examine synergistic interactions and confounding comorbidity effects. Longitudinal animal experiments with real-world dosing and intermittent bacteremia, rather than continuous inoculation, may better recapitulate human exposure.

### Large-scale longitudinal and interventional trials

Although there is some data that supports that periodontal therapy can decrease systemic inflammatory markers and improve endothelial function, many of these studies are small, limited in duration, and use surrogate outcomes [17, 81]. Therefore, large, multicenter, randomized clinical trials are needed that will address the question as to whether or not periodontal therapy can decrease hard endpoints in cardiovascular medicine, including myocardial infarction, stroke, or cardiovascular mortality. Such trials, using standardized biomarker panels, clinical endpoints that are uniform, and sufficiently long-term follow-up, can give definitive results.

Additional value may be gained from embedding biomarker substudies in existing cardiovascular trials (e.g., statin or anti-inflammatory agent trials), and by using adaptive trial designs to evaluate treatment–biomarker interactions. Mendelian randomization approaches may also help clarify causality if suitable genetic instruments for periodontitis or inflammatory markers are available.

### Point-of-care devices and rapid diagnostics

The development of rapid chairside diagnostic assays for biomarkers such as CRP, IL-6, and MPO is facilitated by improvements to biosensors and lateral flow assays [73]. Such devices can be applied in dental offices, primary care, cardiology offices, and emergency rooms to allow cardiovascular risk screening in real-time to promote earlier preventative interventions. Successful acceptance will require regulatory validation, demonstration of an attractive cost–benefit ratio, and incorporation into the clinical workflow.

Among currently available periodontal biomarker technologies, the aMMP-8 chairside assay is one of the most clinically advanced. Unlike total MMP-8 measurements, detection of the active collagenolytic form provides a more specific indicator of ongoing periodontal tissue breakdown. This point-of-care approach has been validated for identifying active periodontal destruction and monitoring treatment response, and it represents an important translational tool linking periodontal diagnostics with systemic inflammatory risk assessment [51, 83]. However, reliable implementation of oral-fluid point-of-care diagnostics requires standardized collection protocols, because variability in salivary flow, sample dilution, and stimulation conditions can significantly affect biomarker measurements.

Further clinical validation studies comparing point-of-care oral biomarker devices with gold-standard laboratory methods across diverse populations and settings remain necessary. In parallel, health economic analyses are needed to determine real-world cost-effectiveness and implementation value. Integration of these technologies into electronic health records and clinical decision-support systems may further enhance their utility by enabling automated risk alerts, referral pathways, and personalized oral-systemic care strategies. Looking ahead, future platforms may combine multiple biomarkers into a single assay and incorporate digital health tools for automated interpretation, longitudinal monitoring, and broader clinical applicability across different ethnic groups, oral health conditions, and systemic comorbidities.

### Interdisciplinary collaboration

The successful translation of biomarker discovery into clinical practice necessitates interdisciplinary partnerships across dentistry, cardiology, epidemiology, and bioengineering. These collaborations are needed for the design of clinically relevant trials, production of multi-biomarker panels, and scale-up of POC technologies. Global consensus frameworks created by organizations such as the European Federation of Periodontology and the American Academy of Periodontology [2, 8], can serve as templates for integrating cross-disciplinary scientific advances and accelerating biomarker uptake in precision cardiovascular medicine. Patient-centered studies and implementation science should explore acceptability, usability, and behavioral impact of oral biomarker screening in routine care.

In the future, integrated oral-systemic health clinics may facilitate risk screening, longitudinal monitoring, and co-management of at-risk patients. Development of consensus protocols and data-sharing networks will accelerate knowledge translation.

## Conclusion

Periodontal biomarkers provide essential mechanistic and diagnostic insight into how local oral inflammation contributes to systemic vascular disease. Evidence supports their role as mediators of inflammation and predictors of cardiovascular events. Advances in omics technologies and artificial intelligence are enabling integration of complex biomarker data into predictive models, while portable POC devices offer real-time assessment of risk at the dental or medical chairside. To translate these discoveries into clinical impact, standardized methodologies, large multicenter trials, and interdisciplinary collaboration across dentistry, cardiology, and bioengineering are imperative. Through validation and technological integration, periodontal biomarkers may evolve from research indicators to practical tools for precision cardiovascular risk assessment, promoting earlier detection and personalized prevention.

## Data Availability

No datasets were generated or analysed during the current study.

## Abbreviations

CRP: C-reactive protein
HSP: Heat-shock protein
IL: Interleukin
LDL: Low-density lipoprotein
LPS: Lipopolysaccharide
MMP: Matrix metalloproteinase
MPO: Myeloperoxidase
NO: Nitric oxide
ROS: Reactive oxygen species
TNF-α: Tumour necrosis factor-α
TLR: Toll-like receptor
